# More cognitive gains from social activity in the oldest-old: evidence from a 10-year longitudinal study

**DOI:** 10.3389/fpsyg.2024.1382141

**Published:** 2024-10-14

**Authors:** Boyu Zhai, Xiaomei Liu, Jiangning Fu, Xinyi Zhu, Juan Li

**Affiliations:** ^1^Center on Aging Psychology, CAS Key Laboratory of Mental Health, Institute of Psychology, Chinese Academy of Sciences, Beijing, China; ^2^Department of Psychology, University of Chinese Academy of Sciences, Beijing, China

**Keywords:** cognitive decline, social activity engagement, trajectory, oldest-old, older adults

## Abstract

**Background:**

Previous research has indicated that engagement in social activities has proven advantageous for diminishing the likelihood of cognitive decline. However, no study has examined whether such cognitive benefits were to a similar extent for the young-old, the old–old, and the oldest-old groups. The purpose of this research was to determine whether aging would have an impact on the changes in cognitive function that would occur in older adults with varying degrees of social involvement.

**Methods:**

The sample for this study comprised 4,481 older adults who participated in the Chinese Longitudinal Healthy Longevity Survey (CLHLS) during the waves spanning from 2008 to 2018. At baseline, participants were classified into the young-old (60–69 years; *M*_age_ = 66.66; *SD* = 1.87), the old–old (70–79 years; *M*_age_ = 74.21; *SD* = 2.82), and the oldest-old (80 years or older; *M*_age_ = 86.46; *SD* = 5.71) groups.

**Results:**

The level of cognitive function decreased as participants aged. Importantly, compared to those lacking social activities, individuals who were got involved in social engagement at baseline had slower rates of cognitive decline over time. Furthermore, compared with the young-old group and the old–old group, the impact of social activity engagement on slowing cognitive decline was more salient for the oldest-old group.

**Conclusion:**

Active engagement in social activities can slow age-related cognitive decline, particularly for the oldest-old group. To preserve cognitive function with aging, attention and resources should be allocated to encourage social activity engagement.

## 1 Introduction

With 13.50% of the population 65 years of age or over, China has the highest percentage of older adults worldwide (National Bureau of Statistics of China, 2021), which is higher than the world average of 9.1% (United Nations, Department of Economic and Social Affairs, Population Division, 2019). A common problem among older adults is the decline in cognitive function (Salthouse, 2009), and such decline can affect many domains, for instance, quality of life (Chen et al., 2020). Therefore, it is essential to identify potential factors that can slow down age-related cognitive decline, so that particular actions can be taken to improve well-being and promote healthy longevity.

One of the main lifetime activities that may be done is engaging in social activities (SA), which has been shown to be helpful for lowering the risk of cognitive decline (e.g., Anatürk et al., 2018; Pugh et al., 2021; Kelly et al., 2017). SA reflect the degree of participation in activities that provide interactions with others within personal, social, or community networks (Levasseur et al., 2010), and it usually can be measured by levels of engagement in facilitator-led group discussions, field trips, and attendance of social groups (Berkman et al., 2000; Fan et al., 2021). In accordance with the cognitive reserve theory (Stern, 2012), our brain can actively resist the adverse effects of age through the concept of cognitive reserve. Cognitive reserve is influenced by life experiences, like participation in SA, and individuals with higher levels of cognitive reserve are better able to cope with age-related brain changes. Research has suggested that engaging in SA may buffer against the adverse effects of aging on cognition by enhancing the brain’s neural connections and cognitive abilities, protecting the individual from cognitive impairment, and replenishing alternative neural pathways when needed (Evans et al., 2018). It is crucial to acknowledge the possibility of the effect of cognition on engaging in SA, such that better maintenance of cognitive ability with aging can lead to higher likelihood of SA participation (Bielak and Gow, 2023; Li et al., 2022). Although we cannot ignore this complex relationship, the main focus of this study was on the contribution of SA engagement to age-related cognitive change.

Consistent with the cognitive reserve theoretical framework, several studies have found that increased SA has a protective impact on cognitive function (Bae, 2021; Brown et al., 2016; Fan et al., 2021). Using the data from a longitudinal sequential project (*M*_age_ = 70.27 years, *SD* = 7.26), Bae (2021) discovered that older adults who engaged in a higher frequency of SA demonstrated elevated scores in cognitive functions, in contrast to those with fewer SA.

However, some studies failed to find the supporting evidence for the protective effect of SA (Aartsen et al., 2002; Iwasa et al., 2012). For instance, Iwasa et al. (2012) identified that there was no significant longitudinal correlation between SA and cognitive decline in Japanese older adults (*M*_age_ = 68.3 years, *SD* = 3.5). Even some studies reported somewhat conflicting results showing that individuals with a higher frequency of SA exhibited poorer cognitive function in comparison to those who did not engage in SA (Lam et al., 2015).

These inconsistent findings suggested that the buffering impact of SA on cognitive decline remains unclear and needs to be clarified. Two competing models, the “differential preservation” and “preserved differentiation” models, have been proposed to explain these discrepancies (Bielak and Gow, 2023). The “differential preservation” model suggests that both active and inactive individuals show cognitive declines over time, but the more active ones would show a lesser degree of change, indicating that cognitive abilities are “differently preserved” as a function of level of activity engagement. Conversely, the “preserved differentiation” model suggests that the more active individuals start at a higher cognitive level, both groups undergo a comparable rate of cognitive decline over the course of time.

One possible factor that could be explored in relation to the two competing models is age, such that different age groups would show different types of preservation, i.e., the young-old (60–69 years), the old–old (70–79 years), and the oldest-old (80+ years). Cognitive aging is a non-linear process, particularly for fluid abilities, which has a relatively stable decline stage during middle adulthood and young-old age and a sharp decline stage during the very late adulthood (Salthouse, 2019). At the relatively stable decline stage, cognitive reserve may not act obviously to slow down the rate of cognitive decline because the brain has not accumulated enough neural burden at this time, which may show the “preserved differentiation.” However, at older age when individuals start to show a sharp cognitive decline, which is when a significant amount of neural burden has been accumulated, the buffering role of cognitive reserve could become active (Amieva et al., 2014). Therefore, the beneficial influence of SA engagement on slowing down cognitive decline might become more obvious and exaggerated with advancing age, thus showing the “differential preservation.”

Very few has examined this moderation effect of age, but some have provided indirect evidence to support this idea. For example, in a study of 110 community-dwelling older adults, Kwak et al. (2020) examined the impact of cognitive reserve on the association between brain gray matter volume and episodic memory performance (*M*_age_ = 72.91, *SD* = 6.38), and focused on whether the effect differed across different age groups. The results showed that, only for the older age group (i.e., *M*_age_ + 1 *SD*), the decline in memory performance was less affected by the gray matter volume shrinkage among individuals with more cognitive reserve. The results suggested that the buffering effect of cognitive reserve may be active only at a relatively older phase of aging when a significant amount of neuropathological burden exists, which also supports the differential preservation model.

In short, little has been done to examine whether the cognitive benefits of SA participation were to a similar extent for different age groups among older adults. Therefore, based on nationally representative longitudinal data, the goal of the current study was to compare the “differential preservation” versus “preserved differentiation” perspective by examining how cognitive function would change over time for older adults with different levels of SA participation (i.e., having SA vs. lacking SA), and whether the degree of change would vary as a function of age. Latent growth curve model (LGCM; Kaplan, 2000) was used to estimate the different trajectories of cognitive function for different age groups, as it can describe individual changes over time using multi-wave data (Duncan et al., 2013). We hypothesized that: (1) compared to those who lacked SA, individuals who engaged in SA at baseline would have lower rates of cognitive decline over time; and (2) based on the two model perspectives, compared with the young-old and the old–old, the beneficial impact of SA engagement on slowing down cognitive decline would be more salient for the oldest-old.

## 2 Methods

### 2.1 Participants

The Chinese Longitudinal Healthy Longevity Survey (CLHLS), which is a prospective cohort study aimed at identifying factors associated with healthy aging, provided the data used in this study (Zeng, 2004). CLHLS surveyed 23 of 31 provinces and covered 85% population. The present study used data from the 2008 (Wave 1), 2011 (Wave 2), 2014 (Wave 3), and 2018 (Wave 4) waves.

**Figure 1:**
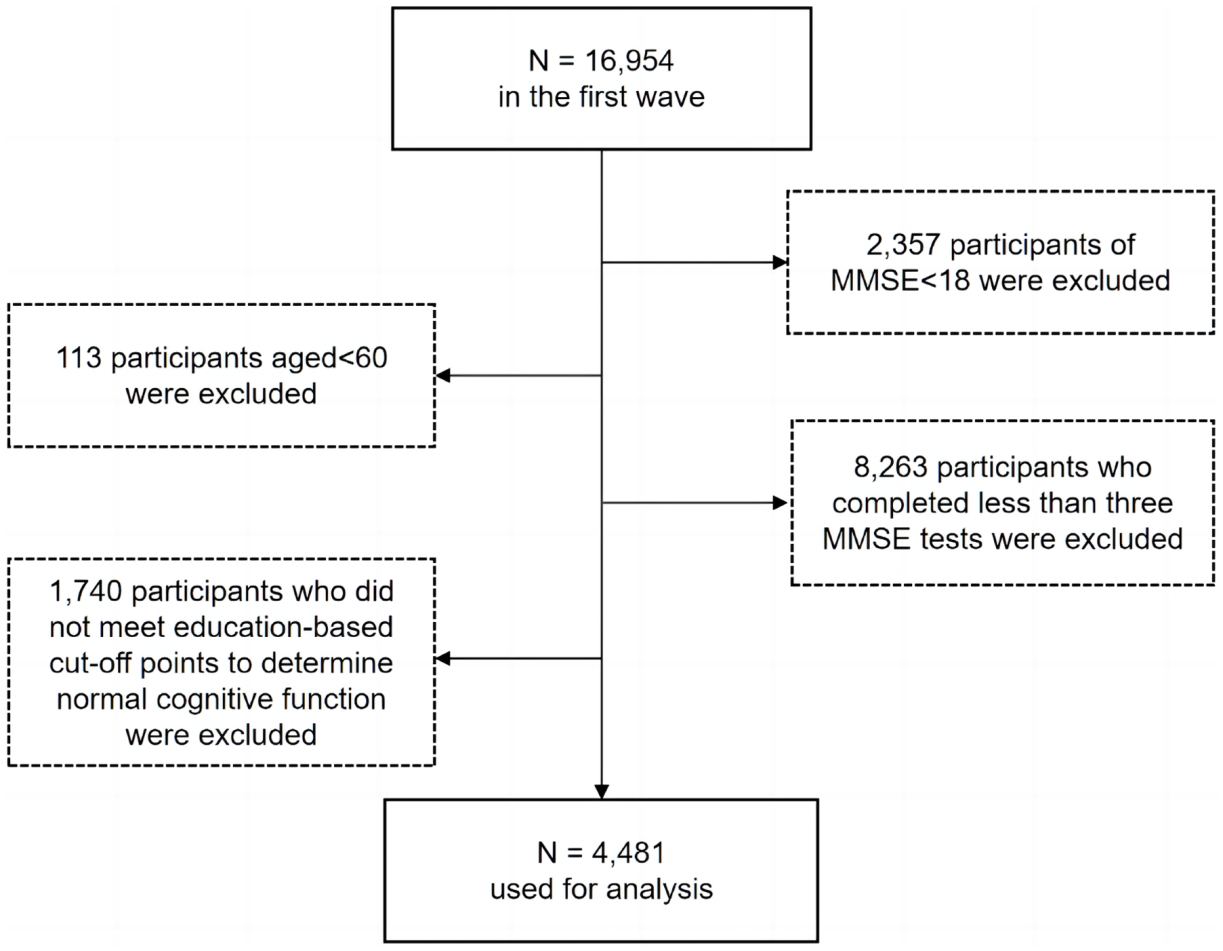
Flow chart of participants.

These were the conditions for inclusion: (1) ≥60 years at baseline; (2) having normal cognitive function at baseline as defined by education-based cut-off points of Mini-Mental State Examination (MMSE) scores (i.e., 18 or higher for illiterate participants, 21 or higher for participants receiving no more than 6 years of education, and 25 or higher for participants receiving more than 6 years education, Zhou et al., 2020); (3) having at least three MMSE tests completed. Thus, as illustrated in the flowchart (Figure 1), the final sample included 4,481 participants. At baseline, participants were classified into the young-old (60–69 years), the old–old (70–79 years), and the oldest-old (80 years or older). The three age groups had 1,199, 1,972, and 1,310 participants, respectively.

Compared to those who were excluded, the eligible participants were more likely to be younger, be better educated, and have better cognitive function and higher levels of SA participation (Supplementary Table S1). Thus, the observed relationships between SA, cognitive function, and age in the current study may be underestimated.

The CLHLS study was approved by the Research Ethics Committees of Peking University and Duke University (IRB00001052-13074). Written informed consent was given by each subject (Zeng et al., 2017).

### 2.2 Measures

#### 2.2.1 Cognitive function

MMSE was used, which was widely used in evaluating global cognitive function (Tsoi et al., 2015). Better cognitive performance was indicated by a higher score, which varied from 0 to 30.

#### 2.2.2 Social activity

The SA level was measured via four aspects, including frequency of participating in organized SA, group leisure-time activities, traveling, and whether participants were still involved in a paid job (Berkman et al., 2000; Fan et al., 2021; Gao et al., 2018). In the original CLHLS survey, each aspect was measured with a single item. Organized SA and group leisure-time activity participation (i.e., cards/mah-jong) were individually rated on a 5-point scale, ranging from “almost everyday” to “never.” The measurement of travel frequency involved querying participants about the number of times they had undertaken journeys in the preceding 2 years. Occupation status was assessed by whether participants were still engaged in a paid job (1 = yes, 0 = no).

We recoded the responses and generated a binary composite score to reflect the level of SA participation (having SA vs. lacking SA). The first step was to recode the responses regardless of frequency, except for the occupation status, which was already in a binary format. For organized SA and group leisure-time activity, the response “never” was coded as 0, whereas others were coded as 1. For traveling, no traveling at all in the past 2 years was coded as 0, whereas any other responses were coded as 1. The second step was to combine the four aspects to generate one composite score, such that participation in at least one of the four categories was considered as having SA and participation in none of them was treated as lacking SA (1 = participation, 0 = non-participation).

Additionally, to consider the complexity of SA participation, we recoded and created a variable to indicate how many categories of SA participants were engaged in (0 = non-participation, 4 = participation in all four categories). The modeling results using this variable were reported as the supplementary analysis.

#### 2.2.3 Covariates

Similar to previous research (e.g., Ji et al., 2020), several covariates were controlled in the model estimation, including demographic variables, health status, functional limitations and depressive symptoms, which were considered to have impacts on cognition. Demographic variables were gender, age, types of residence, current marital status, years of schooling, and financial status. Health status was assessed by the number of diagnosed diseases from a list of 22 prevalent chronic medical conditions that can influence cognitive functions, such as hypertension, diabetes, CVD, epilepsy, cataract, and cancer. Functional limitations are captured by six types number of Activities of Daily Living (ADL) limitations. Five-item scales were used to measure depressive symptoms. A sample item was “Do you often feel anxious or fearful.” Each item received a score between 1 and 5, where a larger number denoted greater depression.

### 2.3 Missing data

Consistent with most longitudinal research, there was data attrition in each collection wave. The missing rate was 1.6% at Wave 1, 3.4% at Wave 2, 19.6% at Wave 3, and 58.4% at Wave 4. We used logistic regression analyses to identify the missing value patterns. Results showed that there were no significant differences between “completers” and “non-completers” on our primary variables, including cognitive function (OR = 0.98, *p* = 0.107) and SA (OR = 1.04, *p* = 0.583). Therefore, the data were missing at random (Little, 1988), and this pattern would not introduce bias into parameter estimates (Enders, 2010).

### 2.4 Data analysis

The various trajectories of cognitive function among participants with and without SA were determined using LGCM (Kaplan, 2000). Full information maximum likelihood (FIML) estimation strategy was used to handle missing data, which produced less biased parameter estimates (Lee and Shi, 2021).

Using the maximum likelihood estimation method, LGCM estimated the intercept (reflecting the initial level of cognitive function) and slope (reflecting the linear change in cognition) of the trajectory. For the slope, factor loadings of 0, 3, 6, 10 were used to reflect the time intervals between waves. SA level was included in the model to predict the intercept and slope. In the main analysis, SA level was treated as a binary variable, and in the supplementary analysis, it was treated as a multiple categorical variable. In parameter estimation, relevant covariates were controlled. Model fit was assessed by the following cut-off criteria (Marsh et al., 2005): the comparative fit index (CFI ≥ 0.90), the Tucker-Lewis index (TLI ≥ 0.90; Byrne, 2012), the root mean square error of approximation (RMSEA ≤0.08; Browne and Cudeck, 1993) and the standardized root mean square residual (SRMR ≤0.08; Hu and Bentler, 1999). All LGCM analyses were conducted using Mplus 7 (Muthén and Muthén, 2017).

## 3 Results

### 3.1 Sample characteristics

Table 1 shows the sample characteristics and age differences for major variables. In general, there were no differences in the locations of living areas (rural vs. urban/town) or financial conditions among the age groups. Compared to younger older adults, older individuals tended to be female, had fewer years of education, were not in married or cohabiting status, participated less in SA, and had poorer cognitive function.

**Table 1:** Descriptive statistics by age groups and age difference for all major variables.

Variables	Total (*n* = 4,481)	Young-old (*n* = 1,199)	Old–old (*n* = 1,972)	Oldest-old (*n* = 1,310)	*p*-value
Age	75.77 (8.41)	66.66 (1.87)	74.20 (2.82)	86.46 (5.71)	<0.001
Male, *n* (%)	2,269 (50.63)	638 (53.21)	1,023 (51.88)	608 (46.41)	=0.001
Years of education	3.05 (3.80)	4.45 (3.98)	2.98 (3.75)	1.88 (3.25)	<0.001
Rural, *n* (%)	2,794 (62.35)	778 (64.89)	1,199 (60.80)	817 (62.37)	=0.071
Income	19,860 (24,383)	19,038 (22,000)	18,986 (24,263)	21,920 (26,450)	=0.001
No financial strain, *n* (%)	3,531 (78.80)	951 (79.32)	1,534 (77.79)	1,046 (79.85)	=0.323
Married/cohabiting, *n* (%)	2,593 (57.87)	925 (77.15)	1,230 (62.37)	438 (33.44)	<0.001
Number of diseases	1.12 (1.32)	1.15 (1.42)	1.23 (1.39)	0.92 (1.08)	<0.001
Number of ADL limitations	0.04 (0.32)	0.02 (0.19)	0.03 (0.31)	0.08 (0.42)	<0.001
Depressive symptoms	11.47 (3.08)	11.00 (2.89)	11.55 (3.08)	11.77 (3.20)	<0.001
MMSE					
Wave 1	27.64 (2.69)	28.65 (1.81)	27.80 (2.42)	26.45 (3.24)	<0.001
Wave 2	26.76 (4.46)	28.25 (2.65)	27.07 (4.07)	24.96 (5.60)	<0.001
Wave 3	26.09 (4.78)	28.05 (2.67)	26.76 (3.66)	23.85 (6.60)	<0.001
Wave 4	25.58 (3.94)	27.71 (2.74)	25.99 (3.16)	21.49 (4.86)	<0.001
Having SA, *n* (%)	1,882 (42.00)	596 (49.71)	838 (42.49)	448 (34.20)	<0.001
Organized SA	4.58 (1.01)	4.46 (1.14)	4.57 (1.99)	4.70 (0.86)	<0.001
Cards/mah-jongg	4.32 (1.33)	4.14 (1.40)	4.32 (1.33)	4.44 (1.25)	<0.001
Traveling	0.22 (1.14)	0.34 (1.67)	0.2 (0.91)	0.13 (0.79)	<0.001
Have a paid job, *n* (%)	283 (6.32)	73 (6.09)	144 (7.30)	66 (5.04)	<0.001

*n* is the sample size. Data are mean (standard deviation), unless otherwise stated. The unit of average annual income is RMB. MMSE, Mini-Mental State Examination. Social activity is a binary composite variable. For organized social activity and cards/mah-jongg, the lower the score, the higher the frequency of participation. Traveling reflects the number of trips within 2 years. ADL, activities of daily living; SA, social activities.

### 3.2 Cognitive change as a function of social activity engagement

The LGCM was fit for the full sample (regardless of age groups) to test our first hypothesis on the overall effect of SA engagement on cognitive change, and then separately for different age groups (i.e., young-old, old–old, and oldest-old) to examine the moderation effect of age. Table 2 shows the model fit for all models to be adequate.

**Table 2:** Model fit for the latent growth curve model in the full sample and different age groups.

Criteria	All participants			Young-old			Old–old			Oldest-old		
	Total	Have SA	No SA	Total	Have SA	No SA	Total	Have SA	No SA	Total	Have SA	No SA
CFI	0.975	0.969	0.975	0.977	1.000	0.951	0.967	0.946	0.980	0.951	0.952	0.963
TLI	0.960	0.953	0.961	0.964	1.051	0.928	0.947	0.917	0.968	0.923	0.926	0.934
RMSEA	0.025	0.025	0.026	0.018	0.000	0.030	0.021	0.025	0.016	0.032	0.030	0.047
SRMR	0.019	0.033	0.022	0.019	0.029	0.032	0.025	0.025	0.025	0.023	0.040	0.044

SA, social activities; CFI, comparative fit index; TLI, Tucker–Lewis index; RMSEA, root mean square error of approximation; SRMR, standardized root mean square residual.

#### 3.2.1 Regardless of age groups

**Figure 2:**
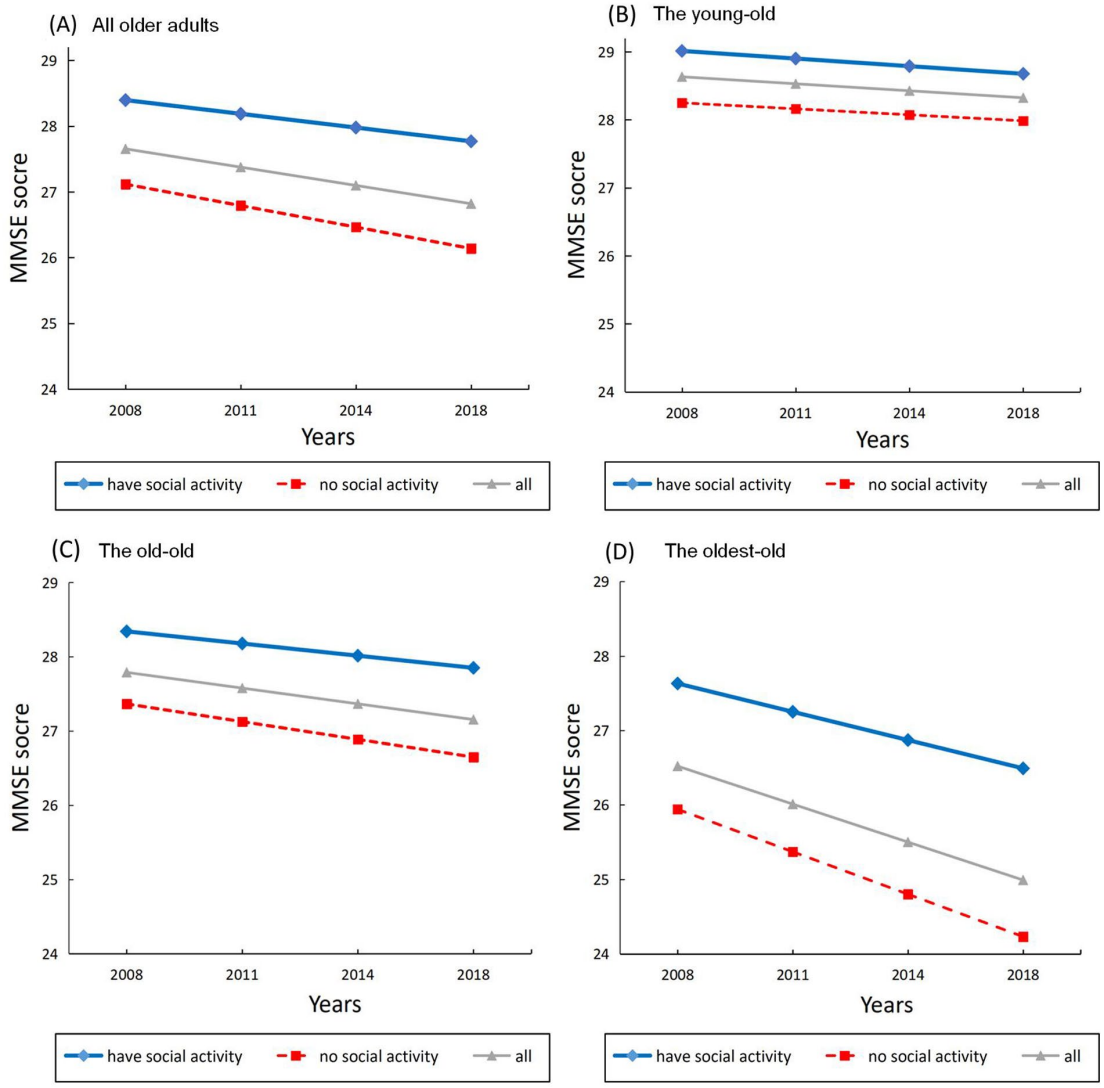
The effects of social activity engagement on estimated developmental trajectories of cognitive function. **(A)** All older adults. **(B)** The young-old group. **(C)** The old–old group. **(D)** The oldest-old group.

As indicated in Figure 2A, after controlling for covariates, baseline individuals who participated in SA had higher levels of initial cognitive function than those who had no SA (*b* = 0.24, *p* < 0.001). Additionally, SA positively impacted the slope of cognitive change (*b* = 0.10, *p* = 0.006), suggesting that older adults who participated in SA at baseline showed a slower rate of cognitive decline over time. Thus, our first hypothesis was supported such that SA engagement had a beneficial impact on slowing down age-related cognitive decline.

Importantly, this beneficial impact of SA engagement was moderated by age (*b* = 0.19, *p* < 0.001). To further examined the moderation effect (i.e., our second hypothesis), we then estimated LGCM growth parameters for different age groups. Parameter estimates for the LGCM in the full sample and different age groups were presented in Table 3.

**Table 3:** Parameter estimates for the latent growth curve model in the full sample and different age groups.

Variables	Total (95% CI)	Have SA (95% CI)	No SA (95% CI)
**All participants**			
Intercept	27.65 (27.57, 27.73)	28.40 (28.31, 28.48)	27.11 (27.00, 27.23)
Slope	−0.28 (−0.30, −0.26)	−0.21 (−0.24, −0.18)	−0.33 (−0.36, −0.29)
**Young–old**			
Intercept	28.63 (28.53, 28.73)	29.01 (28.90, 29.12)	28.25 (28.10, 28.40)
Slope	−0.10 (−0.13, −0.08)	−0.11 (−0.14, −0.08)	−0.09 (−0.13, −0.05)
**Old–old**			
Intercept	27.78 (27.68, 27.88)	28.35 (28.22, 28.47)	27.37 (27.22, 27.51)
Slope	−0.21 (−0.24, −0.18)	−0.17 (−0.21, −0.13)	−0.24 (−0.28, −0.20)
**Oldest–old**			
Intercept	26.51(26.34, 26.69)	27.63 (27.40, 27.85)	25.37 (25.07, 25.66)
Slope	−0.51 (−0.56, −0.45)	−0.38 (−0.45, −0.31)	−0.68 (−0.83, −0.54)

SA, social activities; CI, confidence interval. The intercept reflects the initial level of cognitive function, and the slope reflects the average change corresponding to each incremental year.

#### 3.2.2 Young-old group

The effect of SA was hold for initial intercept of cognitive function (*b* = 0.28, *p* < 0.001). However, Figure 2B shows that the effect of SA was not significant for the slope in the young-old group (*b* = −0.02, *p* = 0.886).

#### 3.2.3 Old–old group

As shown in Figure 2C, similar pattern was yielded such that individuals who participated in SA had better cognition at baseline (*b* = 0.32, *p* = 0.001), but the rate of cognitive decline did not differ between the two activity groups (*b* = 0.03, *p* = 0.496).

#### 3.2.4 Oldest-old group

Figure 2D shows the development trajectory of the oldest-old age group. Adults who participated in SA obtained higher baseline cognitive function scores (*b* = 0.28, *p* < 0.001). And importantly, the rate of cognition decline was slower in SA engagement group compared to the no-activity group (*b* = 0.13, *p* = 0.058), suggesting a significant differential preservation effect.

These results supported our second hypothesis that compared with the young-old and the old–old age groups, the impact of SA engagement on slowing down cognitive decline was more salient for the oldest-old group.

### 3.3 Supplementary analysis

In the full sample, the proportions of engaging in one, two, three, and four categories of SA were 27.76, 10.98, 2.81, and 0.45%, respectively (see Supplementary Table S2 for more details). Due to the very small number of participants engaging in three or four categories of SA and the poor model fitting performance when fitting individual models, we combined them with those participating in two categories of activities for analysis.

To investigate the moderating effect of age, LGCM was fit for the full sample and then individually for each age group. Using the full sample, we found that the interaction between age and the number of SA categories remained significant (*b* = 0.17, *p* < 0.001). Next, the LGCM growth parameters were calculated for different age groups in order to investigate the moderating effect. In both the young-old and old–old age groups, the effect of SA was not significant for the slope (*p*s > 0.05). In the oldest-old group, a greater number of SA categories were associated with a slower decline in MMSE scores (*b* = 0.14, *p* = 0.056). Specifically, the estimated slope for participants engaging in one category of SA is −0.382 (95% CI: −0.472, −0.292), while for those engaging in two or more categories, the estimated slope is −0.370 (95% CI: −0.470, −0.270). Thus, the more categories of SA one participated in, the greater the potential protective effect on age-related cognitive decline. However, given the minimal difference in parameter estimates, this result should be interpreted with caution.

## 4 Discussion

Using a population-based sample, this study examined how cognitive function changed over time for older adults with different levels of SA participation and whether the degree of change varied for different age groups. The results showed that individuals who participated in SA at baseline had slower rates of decline in cognitive function over 10 years, and this protective effect on slowing down cognitive decline was more evident in the oldest-old. This contributed to the literature that the buffering role of SA engagement on cognition can become more active with advancing age.

This study found that compared to those who lacked SA, older adults who engaged in SA had better baseline cognitive function and slower rates of cognitive decline over a 10-year follow-up. Our results supported the beneficial effect of SA, which is consistent with previous studies (e.g., Fan et al., 2021). Based on cognitive reserve theory, individuals who participate in SA can increase their cognitive reserve by gaining neurobiological capital (e.g., neurons). Thus, they can actively cope with age-related pathological changes and maintain cognitive function consequently. It is also essential to note that the effect of SA engagement was hold after adjusting for other factors, especially the proxy for prior cognition as indicated by the level of education. Although more proxies may need to be adjusted in future studies (Bielak and Gow, 2023), this study supported that SA could have a causal beneficial effect on later cognitive abilities.

Importantly, our results showed that age moderated the impact of SA on cognition such that the oldest-old can gain more cognitive benefits from participating in SA. This result was in line with the prior findings (Kwak et al., 2020); that is, the buffering effect of cognitive reserve on the slope of cognitive decline differed across different age groups. In the young-old and old–old age group, the results supported the “preserved differentiation” model, which means that SA engagement has a protective effect on the initial cognitive function rather than the rate of cognitive decline. In contrast, in the oldest-old age groups, the results supported the “differential preservation” model, which means that higher levels of SA engagement not only benefit the initial cognitive function but also slow down the rate of cognitive decline over time. Thus, through our investigation into the association between SA engagement and cognitive decline, the findings implied an integration of diverse perspectives and provided valuable theoretical insights, particularly concerning the role of age in this relationship.

In line with the possible explanations mentioned in the introduction, only in an acute cognitive decline stage during very late adulthood, when enough neural burden has accumulated, SA can become active to slow down the rate of cognitive decline. Another explanation could be that as just entering old age, the young-old are still in the better physical condition and have fewer diseases. They can still engage in a wealth of activities that are beneficial to cognitive function like physical exercise and reading activities (Falck et al., 2019). Thus, maintaining cognitive function may be less dependent on participation in SA. However, with aging, physical factors (such as muscle atrophy, decreased bone density, and chronic diseases) may force individuals to reduce the activities they could accomplish when they were younger. On the other hand, older adults, especially the oldest-old, may still be capable of participating in SA as an important part of daily life. At the same time, engaging in SA requires a wide range of skills, such as language communication, non-verbal communication, everyday memory, and emotional skills. Practicing these abilities can help improve both cognition and well-being. Thus, compared to other age stages, the oldest-old may gain more benefits of SA on cognitive function if they continue to engage in SA.

There are some interesting trends as well. Comparing the baseline cognition for different age groups as a function of SA level, although the oldest-old had the lowest baseline MMSE scores, those who participated in SA visually performed better than the average of the old–old group (cf. Figures 2C,D). This result implied that engaging in SA could probably make people in their 80s perform even better than the average of people 10 years younger. This speculation deserves further investigation.

This study has several limitations. To compensate for the limitations of establishing causality due to the longitudinal design instead of an experimental design, we used baseline SA predicting MMSE score changes over the course of follow-up while controlling for baseline MMSE score, which would largely control for the potential effect of MMSE on SA. Second, the present study extracted four items from the CLHLS survey to assess SA participation level. Although they were not from standardized questionnaires, the measurement is carefully conducted based on theory (Berkman et al., 2000) and relevant research (e.g., Fan et al., 2021). Therefore, the composite score can still capture the core information related to SA participation. Third, the sample analyzed in this study exhibited a higher likelihood of being younger, having higher educational attainment, and displaying superior cognitive function and increased participation in SA compared to those who were excluded. This potential selectivity bias may lead to an underestimation of the relationships between SA and cognitive function. Fourth, this study did not examine the impacts on specific cognitive abilities because the CLHLS survey did not include other relevant cognitive measures besides the MMSE. Future research could include a wider variety of cognitive assessments to explore the complex relationship between SA and cognitive function more comprehensively. Fifth, this study only focused on the influence of baseline SA participation on subsequent cognitive function change. Considering that SA participation may change over time (e.g., due to health status), future research should measure SA at multiple time points to gain a more comprehensive understanding of the dynamic relationship between SA and cognitive function. Finally, our sample consisted of Chinese older adults, and further studies must examine the generalizability of findings to other populations.

Our findings have several implications for practice in reducing the risk of cognitive decline. First, people in their 80s (very later in lifespan) can still benefit from SA, and in fact, they can benefit more than younger older adults. Thus, it is never too late for older adults to engage in SA. Second, promoting social participation of older adults could be integrated into existing training programs aimed at improving cognitive function, thus providing another angle to increase the effectiveness of such programs. Third, more resources should be allocated to provide the oldest-old age group enough opportunities to participate in SA, such as within their families, friends, and communities.

Taken as a whole, this large-scale prospective cohort study found that participation in SA can slow down the rate of age-related decline in cognitive function, particularly for the oldest-old group. These findings highlight that older adults, especially the oldest-old group, can maintain their cognitive health through participating in SA, and this may eventually promote healthy longevity.

## Data Availability

Publicly available datasets were analyzed in this study. This data can be found at: https://opendata.pku.edu.cn/dataverse/CHADS.
