# Supplementary material for: More cognitive gains from social activity in the oldest-old: evidence from a 10-year longitudinal study
Source: Front Psychol. 2024 Oct 14;15:1382141. doi: 10.3389/fpsyg.2024.1382141 (PMC11513381; doi:10.3389/fpsyg.2024.1382141)
Supplement: Supplementary file 1 [file Table_1.DOC]

Table S1

*Baseline Characteristics and Between-group Difference of* *Included and Excluded Participants.*

| Variables | Total  (*n*=16,954) | Included  (*n*=4,481) | Excluded  (*n*=12,470) | *p* value |
| --- | --- | --- | --- | --- |
| Age | 86.85 (11.97) | 75.77 (8.41) | 90.82 (10.46) | < 0.001 |
| Male, *n* (%) | 7,252 (42.77) | 2,269 (50.63) | 4,983 (39.95) | < 0.001 |
| Years of education | 2.06 (3.43) | 3.05 (3.80) | 1.70 (3.21) | < 0.001 |
| Rural, *n* (%) | 10,293 (60.71) | 2,794 (62.35) | 7,499 (60.12) | = 0.009 |
| Income | 22,593 (26,380) | 19,860 (24,383) | 23,577 (26,995) | < 0.001 |
| No financial strain, *n* (%) | 13,102 (77.28) | 3,531 (78.80) | 9,571 (76.73) | = 0.005 |
| Married/cohabiting, *n* (%) | 5,251 (30.97) | 2,593 (57.87) | 2,658 (21.31) | < 0.001 |
| Number of diseases | 1.06 (1.22) | 1.12 (1.32) | 1.07 (1.23) | = 0.065 |
| Number of ADL limitations | 0.64 (1.50) | 0.04 (0.32) | 0.87 (1.68) | < 0.001 |
| Depressive symptoms | 12.30 (3.04) | 11.47 (3.08) | 12.60 (2.97) | < 0.001 |
| MMSE  Social activity | 23.50 (7.00) | 27.64 (2.69) | 22.03 (7.47) | < 0.001 |
| Organized social activity | 4.75 (0.80) | 4.58 (1.01) | 4.81 (0.70) | < 0.001 |
| Cards/mah-jongg | 4.61 (1.06) | 4.32 (1.33) | 4.71 (0.91) | < 0.001 |
| Traveling | 0.11 (0.76) | 0.22 (1.14) | 0.08 (0.56) | < 0.001 |
| Have a paid job, *n* (%) | 757 (4.47) | 283 (6.32) | 474 (3.80) | = 0.065 |

*Note.* *n* is the sample size; Data are mean (standard deviation), unless other stated. The unit of average annual income is RMB. MMSE = Mini-Mental State Examination. For organized social activity and cards/mah-jongg, the lower the score, the higher the frequency of participation. Traveling reflects the number of trips within two years. ADL = activities of daily living.
