# Supplementary material for: More cognitive gains from social activity in the oldest-old: evidence from a 10-year longitudinal study
Source: Front Psychol. 2024 Oct 14;15:1382141. doi: 10.3389/fpsyg.2024.1382141 (PMC11513381; doi:10.3389/fpsyg.2024.1382141)
Supplement: Supplementary file 2 [file Table_2.DOCX]

Table S2

*Number of Categories for Social Activities Participation by Age Groups.*

|  | Total  (*n =* 4,481) | Young-old  (*n =* 1,199) | Old-old  (*n =* 1,972) | Oldest-old  (*n =* 1,310) |
| --- | --- | --- | --- | --- |
| None | 2599 (58.00) | 603 (50.29) | 1134 (57.51) | 862 (65.80) |
| One | 1244 (27.76) | 381 (31.78) | 555 (28.14) | 308 (23.51) |
| Two | 492 (10.98) | 162 (13.51) | 213 (10.80) | 117 (8.93) |
| Three | 126 (2.81) | 46 (3.84) | 60 (3.04) | 20 (1.53) |
| Four | 20 (0.45) | 7 (0.58) | 10 (0.51) | 3 (0.23) |

*Note.* *n* is the sample size. Data are *n* (%)
